# DICER Inactivation Identifies Pancreatic β-Cell “Disallowed” Genes Targeted by MicroRNAs

**DOI:** 10.1210/me.2015-1059

**Published:** 2015-06-03

**Authors:** Aida Martinez-Sanchez, Marie-Sophie Nguyen-Tu, Guy A. Rutter

**Affiliations:** Section of Cell Biology and Functional Genomics, Division of Diabetes, Endocrinology and Metabolism, Department of Medicine, Imperial College London, London W12 0NN, United Kingdom

## Abstract

Pancreatic β-cells are the body's sole source of circulating insulin and essential for the maintenance of blood glucose homeostasis. Levels of up to 66 “disallowed” genes, which are strongly expressed and play housekeeping roles in most other mammalian tissues, are unusually low in β-cells. The molecular mechanisms involved in repressing these genes are largely unknown. Here, we explore the role in gene disallowance of microRNAs (miRNAs), a type of small noncoding RNAs that silence gene expression at the posttranscriptional level and are essential for β-cell development and function. To selectively deplete miRNAs from adult β-cells, the miRNA-processing enzyme DICER was inactivated by deletion of the RNase III domain with a tamoxifen-inducible Pdx1CreER transgene. In this model, β-cell dysfunction was apparent 2 weeks after recombination and preceded a decrease in insulin content and loss of β-cell mass. Of the 14 disallowed genes studied, quantitative RT-quantitative real-time PCR revealed that 6 genes (*Fcgrt*, *Igfbp4*, *Maf*, *Oat*, *Pdgfra*, and *Slc16a1*) were up-regulated (1.4- to 2.1-fold, *P* < .05) at this early stage. Expression of luciferase constructs bearing the 3′-untranslated regions of the corresponding mRNAs in wild-type or DICER-null β-cells demonstrated that *Fcgrt*, *Oat*, and *Pdgfra* are miRNA direct targets. We thus reveal a role for miRNAs in the regulation of disallowed genes in β-cells and provide evidence for a novel means through which noncoding RNAs control the functional identity of these cells independently of actions on β-cell mass.

Diabetes mellitus currently affects more than 382 million individuals worldwide, a figure predicted to increase to >590 million by 2035 (1). Pancreatic β-cells are the sole source of circulating insulin in humans, and impaired secretion of the hormone, which is absolute in type 1 diabetes and relative in type 2 diabetes, is ultimately responsible for the emergence of the frank disease. In healthy individuals, β-cells respond to increased levels of blood glucose with enhanced uptake and oxidative metabolism of the sugar. Elevations in cytosolic ATP/ADP ratios, the closure of ATP-sensitive K^+^ channels (K_ATP_), and Ca^2+^ entry through voltage-gated Ca^2+^ channels then trigger the release of the stored hormone (2). Additional coupling mechanisms, largely independent of K_ATP_ channels, also further amplify the effects of glucose (2–4).

Although the expression of key β-cell glucose sensors, including the glucose transporter GLUT2 (*SLC2A2*) (4) and glucokinase (*GCK*), driven by a cluster of β-cell enriched transcription factors, is essential for normal glucose-stimulated insulin secretion (GSIS), our work (5, 6) and that of others (7–9) have identified a group of ∼66 housekeeping genes whose expression is unusually low in the β-cell. These “disallowed” (or “forbidden”) genes, of which 11 were common to the 2 studies above (5), include the lactate/pyruvate (monocarboxylate) transporter MCT-1, encoded by *Slc16a1*, as well as *Ldha.* Up-regulation of the human analog of the former is observed in cases of exercise-induced hyperinsulinism (10), in which activating mutations in the *SLC16A1* promoter lead to the expression of MCT-1 in the β-cell plasma membrane. This allows muscle-derived pyruvate to stimulate mitochondrial oxidative metabolism and hence the release of insulin (11).

MicroRNA (miRNAs) control several aspects of β-cell development and function. Thus, in an early study, Poy et al (12) demonstrated that miR-375, which was highly expressed in β-cells, regulated the expression of myotrophin to control exocytosis. Later studies have shown that specific miRNAs might affect insulin production (13–17), exocytosis (18, 19), growth (20), or apoptosis (21, 22). Depletion of *Dicer* (therefore disrupting miRNA maturation) early in pancreas development resulted in gross defects in all pancreatic lineages and pancreas agenesis (23), whereas disruption only in β-cells during embryonic progression led to defective insulin secretion, β-cell mass reduction, and overt diabetes mellitus (24, 25). Not surprisingly, variations in miRNA expression have been observed during the development of both type 1 and type 2 diabetes and in mouse models of diabetes (26).

The mechanisms responsible for the control of the disallowed genes are as yet largely unclear. In mouse β-cells, *Slc16a1* and *Acot7* are also both subject to control via histone methylation (27, 28). Repression by the winged-helix transcription factor *RFX6*, important for normal β-cell development and function (29), provides a further mechanism for gene silencing (30). Although its role in the control of other disallowed genes has not been examined, DNA methylation is not involved in the control of *Slc16a1* (31).

We have previously shown that miRNAs are involved in the control of *Slc16a1* (MCT-1) (31). Thus, miR-29a and miR-29b target *Slc16a1* mRNA directly. Whether other miRNAs bind to further members of the disallowed gene family is unclear. To address this question systematically, we have therefore explored the impact of deleting DICER highly selectively in the β-cell in adult mice. By preventing the processing of pre-miRNAs, this approach is expected to reveal those mRNAs targeted by mature miRNAs in these cells. Previous studies in which DICER was ablated in β-cells have involved a variety of different approaches and *Cre* deleter strains, including PdxCre (23), which catalyzes recombination in all pancreatic endocrine cell lineages (32), RIP2Cre (24, 25), which deletes in β-cells and, to a substantial degree, in the brain (33), and RIP2CreER (16), which allows more selective deletion in the adult β-cell, with some recombination in the brain. Deletion in neurogenin 3 (NGN3)-positive endocrine precursors has also been used (34). Compared with the deleter strains above, Pdx1CreER, which also allows tamoxifen-controlled recombination in adult mice, provides more selective deletion in the adult β-cell vs brain (with recombination largely restricted to the hypothalamus) at low tamoxifen dosages (35) and has therefore been deployed here.

Previous studies observed up-regulation of transcriptional repressors (16), which contributed to a strong reduction in insulin expression in *Dicer*-null adult β-cells. Nevertheless, no changes in the expression of transcription factors essential for β-cell function were detected, and disallowed gene expression was not interrogated. Here, we describe the functional changes preceding loss of insulin content and β-cell mass, which are associated with up-regulation of β-cell disallowed genes.

## Materials and Methods

### Generation of mice lacking *Dicer* selectively in pancreatic β-cells

Mice homozygous for floxed alleles of the *Dicer* gene (C57BL/6 background) (36), kindly provided by Professor Matthias Merkenschlager (MRC Clinical Sciences Centre, Imperial College), were crossed with PdxCreER mice, provided by Professor D. Melton (Harvard University) (28), expressing Cre-ER under the control of the mouse Pdx1 promoter (C57BL/6 background). The resulting heterozygous mice were subsequently crossed with siblings to generate βDicer-null mice (Dicer^fl/fl^, Cre-ER positive, heterozygous). βDicer-null mice were bred with Dicer^fl/fl^ to generate littermate controls.

### Mouse maintenance

Animals were housed 2 to 5 per individually ventilated cage in a pathogen-free facility with a 12-hour light/dark cycle and had free access to standard mouse chow diet. All in vivo procedures described were performed at the Imperial College Central Biomedical Service and approved by the UK Home Office Animals Scientific Procedures Act, 1986 (HO License PPL 70/7349). Five doses of 100 μL of tamoxifen (Sigma-Aldrich), 20 mg/mL dissolved in corn oil (Sigma-Aldrich), were administered by intraperitoneal injection to 7- to 8-week-old mice. Where relevant, the sex of the animals is indicated in the figure legends.

### Intraperitoneal glucose tolerance test and intraperitoneal insulin tolerance test

After tamoxifen injections, βDicer-null mice and control littermates were fasted overnight intraperitoneal glucose tolerance test (IPGTT) or for 5 hours intraperitoneal insulin tolerance test (IPITT) and glucose (1 g/kg, IPGTT) or insulin (NovoRapid; 0.75 U/kg) were administered intraperitoneally. Glucose levels were measured from tail blood samples using an Accu-Chek Aviva glucometer preceding the glucose/insulin administration and 15, 30, 45, 60, 90, and 120 minutes after the injection.

### Isolation and analysis of mouse islets

Islets were isolated by digestion with collagenase as described previously (37). Islets from βDicer-null and control mice were allowed to recover from digestion overnight in culture medium (RPMI 1640 medium containing 11.1 mM glucose, 10% fetal bovine serum, and l-glutamine) and assayed for insulin secretion as described elsewhere (38) using a homogeneous time-resolved fluorescence-based (HTRF) insulin assay (CisBio) in a PHERAstar reader (BMG Labtech), following the manufacturer's guidelines.

### RNA extraction, RT, and quantitative real-time PCR (qPCR)

Total RNA, including small RNAs, was extracted from islets (50–200) isolated from βDicer-null mice and their wild-type controls, 2 weeks after tamoxifen injections, using TRIzol according to the manufacturer's instructions. RNA (100–500 ng) was reverse transcribed using the High-Capacity cDNA reverse transcription kit (Life Technologies) including random primers. Real-time PCR followed, using a SYBR Green PCR master mix (Life Technologies) and specific primers used for each of the studied genes. The sequences of the primers are indicated in Table 1.

**Table 1.** Primers Used for RT-qPCR

Gene	Forward Primer (5′ → 3′)	Reverse Primer (5′ → 3′)
*Ins2*	CGTGGCTTCTTCTACACACCC	AGCTCCAGTTGTGCCACTTGT
*Dicer*	TTTTGCACGTACCCTGATGCT	CAGTTGCTGACCTTTTTGCTTCT
*Cxcl12*	CCCTGCCGGTTCTTCGA	CAGCCGTGCAACAATCTGAA
*Cd302*	GCGATGGAAGGGTCCAGAT	GAAAGTTGCATCATCAGTGTCATAGA
*C1qbp*	AAGAACAGGAGCCAGAACTGACA	TGCCATCAGTCTTGGTAACTTCA
*Fcgrt*	GCCTGGTTCTCTAGCTCTGTAATTAATT	GACAGGAGTGTTCCTCTGTGAACTT
*Igfbp4*	CGGAGCTGTCGGAAATCG	TTGAAGCTGTTGTTGGGATGTT
*Ldha*	ATGAAGGACTTGGCGGATGA	ATCTCGCCCTTGAGTTTGTCTT
*Lmo4*	TTACACCAAGAGCGGCATGA	CCGCTATTCCCAAATAACCTAATG
*Ndgr2*	CCAACACGCACCCAACCT	TCTCGGCGGTTGTTGTAGCT
*Maf*	CCCCTTGGCCCTGAACA	TCCCTCCCCAATTCAAAAGA
*Oat*	AGGGAAAGGGTTGCTAAATGC	CGCAGGCACACCTTCCA
*Smad3*	CCTCCTGGCTACCTGAGTGAA	TTTGGAGAACCTGCGTCCAT
*Slc16a1*	GCTTGGTGACCATTGTGGAAT	CCCAGTACGTGTATTTGTAGTCTCCAT
*Tns1*	CTGCCCCTTGCGTTCCT	ACTTCCAACCCGGCAGTCT
*Pdgfra*	GACCCTGTTCCAGAGGAGGAA	TTCCGAAGTCTGTGAGCTGTGT
*Sox6*	GACAGCGTTCTGTCATCTCAGCAA	CGTTCCGGGGTTCCAAAAGTAACA
*Slc2a2*	GCAACTGGGTCTGCAATTTTG	CAAGGAAGTCCGCAATGTACTG
*Gck*	TGGTGGATGAGAGCTCAGTGAA	CATGTACTTTCCGCCAATGATC
*Abcc8*	GCCTACGCATCTCAGAAACCA	CCATCTTGTACCTTTGCTTATTGAAG
*Kcnj11*	CACGGCGGGATAAGTCTACCT	AATCATTTGCCCCCTTCTTGT
*Pdx1*	CCAAAGCTCACGCGTGGA	TGTTTTCCTCGGGTTCCG
*Nkx6.1*	GCCTGTACCCCCCATCAAG	GTGGGTCTGGTGTGTTTTCTCTT
*NeuroD*	AACCTTTTAACAACAGGAAGTGGAA	CTGAGGCTCGCCCATCAG
*Mafa*	CTTCAGCAAGGAGGAGGTCATC	CGTAGCCGCGGTTCTTGA
*Rfx6*	TTGGTGCCCGCGTGAT	AGATGGAAAGAGCCAAAACTTGA
*Pax6*	GCACATGCAAACACACATGAAC	GGTGAAATGAGTCCTGTTGAAGTG

For miRNA detection, 100 ng of RNA was reverse transcribed with a TaqMan miRNA reverse transcription kit (Life Technologies), including the appropriate miRNA-specific primer (Life Technologies), using up to 3 primers per reaction simultaneously. qPCR was performed with a TaqMan PCR master mix (Life Technologies) and the relevant miRNA-specific TaqMan probe (Life Technologies).

### Plasmid generation and transfection of mouse islets

Mouse *Pdgfra*, *Oat*, *Fcgrt*, and *Igfbp4* 3′-untranslated regions (UTRs) were amplified by PCR using specific oligonucleotides (sequences can be provided upon request) from mouse islet cDNA and inserted into the pGEM-T vector (Promega). The sequences were then excised from pGEM-T and subcloned into the pmirGLO Dual-Luciferase miRNA Target Expression Vector (Promega).

Mouse islets (100) were hand-picked and transfected using the Neon Transfection system (Life Technologies), following the manufacturer's instructions. In brief, islets were resuspended in 100 μL of resuspension buffer R together with 5 μL of the appropriate DNA and exposed to 2 pulses of 30 ms at 950 V. Twenty-four hours later, islets were lysed, and luciferase activity was determined with the Dual-Glo Luciferase Assay System (Promega) in a BMG Labtech luminometer.

### Immunohistochemistry and apoptosis assay

Isolated pancreata were fixed in 10% (v/v) formalin for 24 hours and embedded in paraffin wax. Sections (5 μm) were cut and fixed on Superfrost slides. Slides were permeabilized as detailed in Ref. 38 and blotted with the following primary antibodies: antiguinea pig insulin (1:200; Dako) and antirabbit glucagon (1:100; Santa Cruz Biotechnology). Slides were visualized using an Axiovert 200M microscope (Zeiss) with Alexa Fluor 488 goat antiguinea pig IgG and with Alexa Fluor 568 donkey antirabbit IgG (Invitrogen). For examination of apoptosis, terminal deoxynucleotidyl transferase dUTP nick-end labeling (TUNEL) assays were performed on the above sections using a DeadEnd Fluorometric TUNEL system kit (Promega) according to the manufacturer's instructions. ImageJ software (Wayne Rasband, National Institute of Mental Health) was used to calculate the mean intensity of insulin staining in the insulin-positive area, β- and α-cell mass, and the number of TUNEL-positive cells of all visible islets. For β-cell mass estimation by immunocytochemistry, we determined the percentage of pancreatic surface that was insulin positive, as measured in whole pancreas sections separated by 25 μm in the z-axis.

### Quantification of pancreatic insulin content

After fasting of mice for 5 hours, pancreata were surgically removed, divided into 2 halves, weighed, and homogenized using a Polytron homogenizer in 5 mL of acidic ethanol (1.5% HCl and 70% EtOH). After overnight incubation under rotation at 5°C, the samples were further homogenized by sonication. After another overnight incubation, samples were centrifuged at 2000 rpm for 15 minutes at 4°C, and the aqueous solution was transferred to fresh tubes. Samples were neutralized with equal volumes of 1 M Tris-HCl (pH 7.2). Appropriate dilutions (1:100–1:1000) were assayed for insulin concentration using an HTRF insulin assay in a PHERAstar reader, following the manufacturer's guidelines. Appropriate calculations were performed to estimate the total amount of insulin per pancreas (nanograms per milligram).

### Statistical analysis

GraphPad Prism 6.0 was used for statistical analysis. Statistical significance was evaluated by the two-tailed paired Student *t* test and one- or two-way ANOVA with a Bonferroni post hoc test when appropriate. All data are shown as means ± SEM. *P* values of < .05 were considered statistically significant.

## Results

### Deletion of *Dicer* using Pdx1CreER leads to glucose intolerance and hyperglycemia

**Figure 1.**
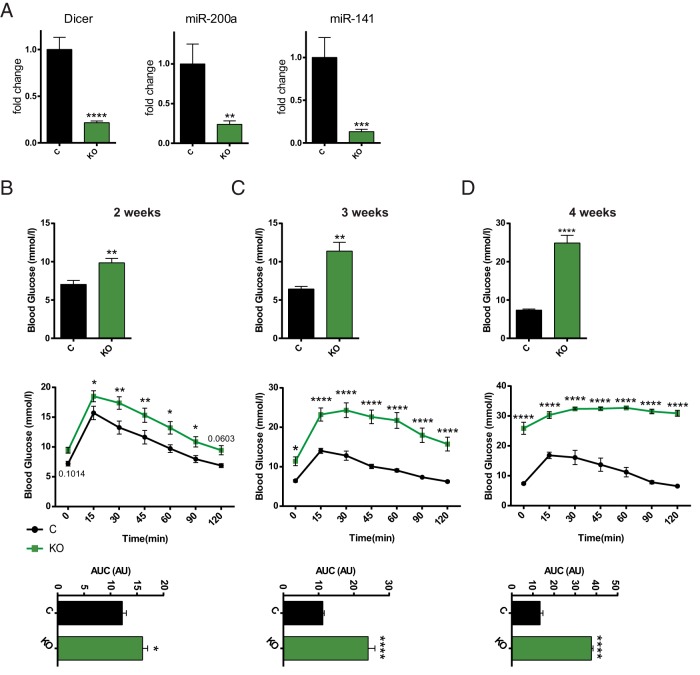
β-Cell–specific deletion of DICER leads to glucose intolerance and hyperglycemia. βDicer-null (KO, 7- to 8-week-old) or control Dicer^flox/flox^ (C) mice were injected with 2 mg of tamoxifen for 5 consecutive days. A, Two weeks after the last injection, total RNA was extracted from isolated islets and RT-qPCR used to evaluate the expression of the indicated genes and miRNAs (n = 9–13 mice/genotype). B–D, Glycemia after overnight fasting (top panels) and glucose tolerance (middle and bottom panels) were measured in βDicer-null (KO) or littermate controls (C) 2 (B), 3 (C), or 4 weeks (D) after tamoxifen injection. The area under the curve (AUC) is shown in the bottom panels: B, n = 5 male and 4 female and 7 male and 8 female C and KO mice, respectively; C and D, n = 2 male and 5 female and 5 male and 4 female C and KO mice, respectively. All mice were 7 to 8 weeks old at the start of the injections. In D, glycemia values at time points 15 to 120 for 4 KO mice and 30 to 90 for an additional 2 KO mice were out of range for glucometer testing. In those cases, glycemia was assigned a value of 33 (maximum). *, *P* < .05; **, *P* < .01; ****, *P* < .0001; Student *t* test (A; glycemia and area under the curve) and two-way ANOVA Fisher least significant difference test (glucose tolerance test).

DICER is essential for miRNA processing, and consequently its ablation results in simultaneous loss of almost the entire “miRNome,” with just a few miRNAs excepted (39). To assess miRNA function specifically in the adult β-cell and to avoid the effects that miRNA absence would have during the maturation of Pdx1^+^ progenitors (23), we bred animals bearing floxed alleles of *Dicer* to mice carrying the Pdx1CreER transgene (28), generating PdxCreER^TM^:Dicer^lox/lox^ mice (βDicer-null). Activation of *Cre* and recombination at *lox*P sites flanking an essential RNase III domain (36) is thus expected to lead to elimination of functional DICER selectively in β-cells upon tamoxifen administration in adult animals. Tamoxifen (2 mg/kg) was administered to 7- to 8-week-old βDicer-null mice or to control Dicer^lox/lox^ littermates. As expected, expression levels of *Dicer* mRNA (containing the targeted exons) and of 2 abundant and β-cell enriched miRNAs, miR-200a and miR-141 were each substantially (>80%) reduced in βDicer-null mouse islets (Figure 1A). Also as expected, *Dicer* expression remained unchanged in the hypothalamus and liver of these mice (Supplemental Figure 1A). In line with previous studies of *Dicer* deletion in the β-cell (16, 25), βDicer-null mice displayed progressive increases in fasting glycemia and deterioration in glucose tolerance from 2 weeks after the end of the treatment (Figure 1B), becoming overtly diabetic by 3 weeks (Figure 1C). Fasting glycemia was exceptionally high (∼30 mmol/L) 4 weeks after injection (Figure 1D). The observed phenotype was independent of the sex of the animals studied (Supplemental Figure 2, A–F). No significant differences in weight were observed 2 and 3 weeks after the tamoxifen treatment, although βDicer-null mice were significantly lighter 4 weeks after the injections, after the onset of frank diabetes (Supplemental Figure 2G). Interestingly, blood glucose levels did not decrease to the same level in βDicer-null animals as in controls after insulin injection (Supplemental Figure 2H). Thus, a change in insulin sensitivity cannot be excluded in these animals, although definitive hyperinsulinemic-euglycemic clamp studies will be necessary to address this question in the future. As expected, glycemia remained unchanged vs that in control mice in the absence of tamoxifen administration (Supplemental Figure 3).

### Impaired GSIS precedes changes in β-cell mass and insulin content upon β-cell–specific *Dicer* deletion

**Figure 2.**
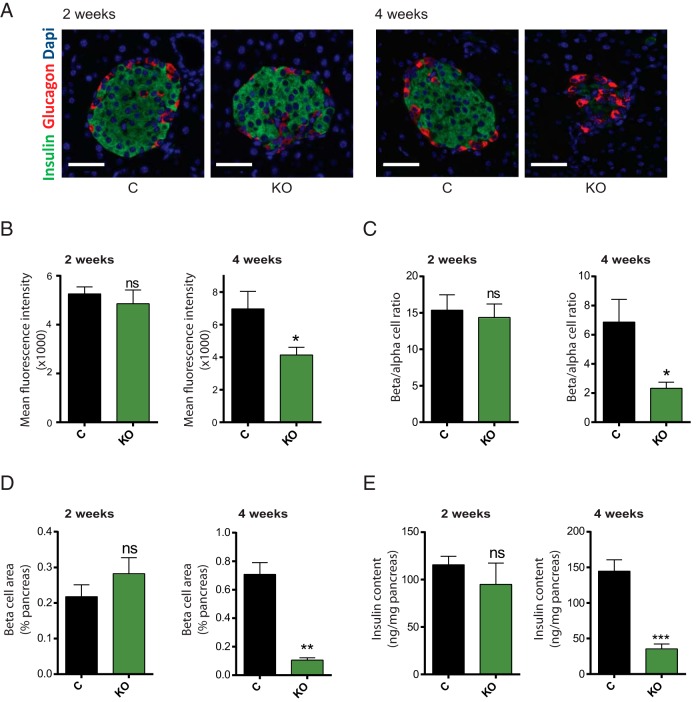
β-Cell mass and insulin content are reduced in βDicer-null islets. Pancreata from βDicer-null (KO) or littermate controls (C), 2 or 4 weeks after tamoxifen administration, were fixed and subjected to immunocytochemical analysis for insulin and glucagon, as indicated. A, representative islets. Dapi, 4′,6-diamidino-2-phenylindole. B, To estimate islet insulin content, ImageJ software was used to quantify fluorescence intensity of insulin staining (per insulin positive surface). C, β- and α-cell masses are presented as the β-cell/α-cell ratio and correspond to quantification of insulin-positive area/glucagon positive area ratio. D, β-Cell mass is presented as a percentage of the pancreatic surface and corresponds to quantification of the insulin-positive area per pancreas area quantified in whole pancreas sections. n = 3 mice/genotype; 5 pancreas sections per animal were used, evenly separated by 25 μm. Scale bar corresponds to 40 μm. E, Pancreatic insulin was extracted from whole pancreas in acidic ethanol and measured using an HTRF insulin assay (n = 3–6 mice/genotype). *, *P* < .05; **, *P* < .01; ***, *P* < .001, Student's *t* test; ns, not significant.

**Figure 3.**
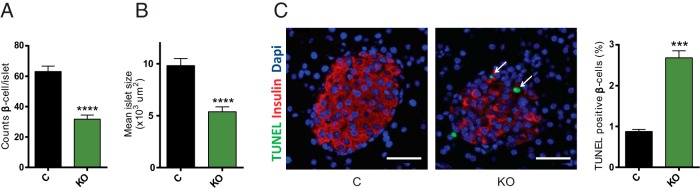
β-Cell apoptosis is increased in βDicer-null islets 4 weeks after tamoxifen injections. Pancreata from βDicer-null (KO) or littermate controls (C) 4 weeks after tamoxifen administration were fixed and subjected to immunocytochemical analysis for insulin and TUNEL, as indicated. A and B, ImageJ software was used to quantify the number of insulin-positive (β-cells) per islet (A) and islet size (B). n = 3 mice/genotype; 5 pancreas sections per animal were used, evenly separated by 25 μm. All the islets present in each slide (15–50) were included in the analysis. C, ImageJ software was used to quantify the number of TUNEL-positive cells, expressed as a percentage of insulin-positive β-cells. Representative islets are shown. Dapi, 4′,6-diamidino-2-phenylindole. n = 3 mice/genotype and 2 pancreas sections per animal were used (100 μm apart). A total of 1710 (βDicer null) and 8915 (control) β-cells were counted. Scale bar corresponds to 40 μm. ***, *P* < .001; ****, *P* < .0001; Student's *t* test.

To understand the cause of the observed hyperglycemia and glucose intolerance, we next assessed, using immunocytochemistry (Figure 2A), insulin expression in β-cells (mean intensity of insulin staining; Figure 2B), the ratio of β-cells/α-cells (Figure 2C), and β-cell mass (Figure 2D) as determined from the percentage of the pancreatic surface occupied by insulin-positive structures. The total pancreatic insulin content was also assessed in pancreatic homogenates (Figure 2E). Whereas none of the above parameters was significantly affected in βDicer-null mice 2 weeks after tamoxifen treatment, each was sharply reduced at 4 weeks posttreatment (Figure 2, B–E). A substantial reduction in the number of β-cells per islet was also observed 4 weeks after tamoxifen in DICER-null islets (Figure 3A), which led to a considerable reduction in the average islet size (Figure 3B). Apoptosis, as assessed by the TUNEL assay, was also markedly increased in β-cells from βDicer-null islets 4 weeks after injection (Figure 3C), whereas, as expected, no differences were observed after only 2 weeks (Supplemental Figure 4). These results suggest that apoptosis at least partially contributes to the observed decreases in β-cell mass at 4 weeks.

**Figure 4.**
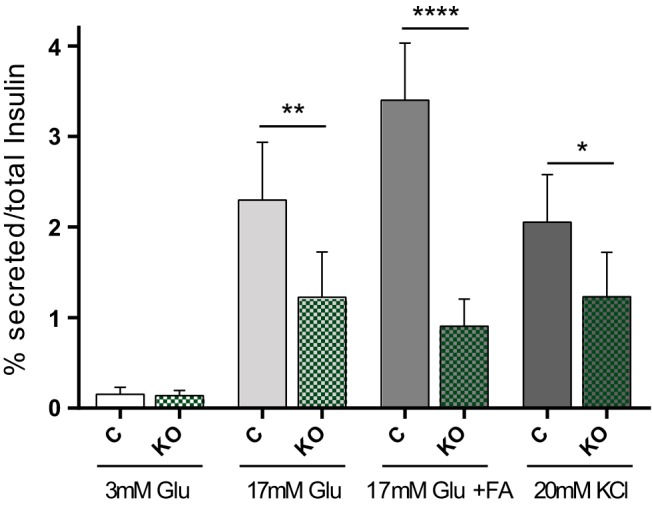
GSIS is markedly impaired in βDicer-null islets early after tamoxifen injections. Insulin secretion in response to 30 minutes exposure to low or high glucose (Glu), alone or in combination with 0.5 mM mixed fatty acids (FA) (3:1 palmitate/oleic acid) and KCl (20 mM) in islets from control (C) or βDicer null (KO) mice, 2 weeks after tamoxifen injection, was measured using an HTRF-based assay. Incubations were performed in duplicate and involved 8 islets/tube (n = 3 mice/genotype). Data are presented as the percentage of insulin secreted vs total insulin content. *, *P* < .05; **, *P* < .01; ****, *P* < .0001; matched two-way ANOVA, Bonferroni test.

The above result indicated that, 2 weeks after tamoxifen injection, the compromised glucose tolerance observed in the βDicer-null mice could not be explained by changes in β-cell mass or insulin content. Therefore, we wondered whether the capacity of islets to respond functionally to high glucose with insulin secretion may be impaired. Accordingly, we isolated islets from βDicer-null and control mice and assessed GSIS in vitro. This approach revealed that, at this early stage, GSIS was already markedly (∼50%) inhibited (Figure 4). Moreover, insulin release in response to high glucose plus fatty acids was even more markedly (>75%) impaired in βDicer-null mice. Depolarization (KCl)-induced secretion was also significantly, although less strongly, inhibited, arguing for effects on both early and late events in glucose signaling and exocytosis. As anticipated, the insulin content of the islets used in these experiments was equal in the 2 genotypes (Supplemental Figure 5). These data thus demonstrate that a marked deterioration in β-cell function precedes the loss of β-cell mass after miRNA depletion from the mature β-cell.

### Disallowed genes are up-regulated in βDicer-null mice

**Figure 5.**
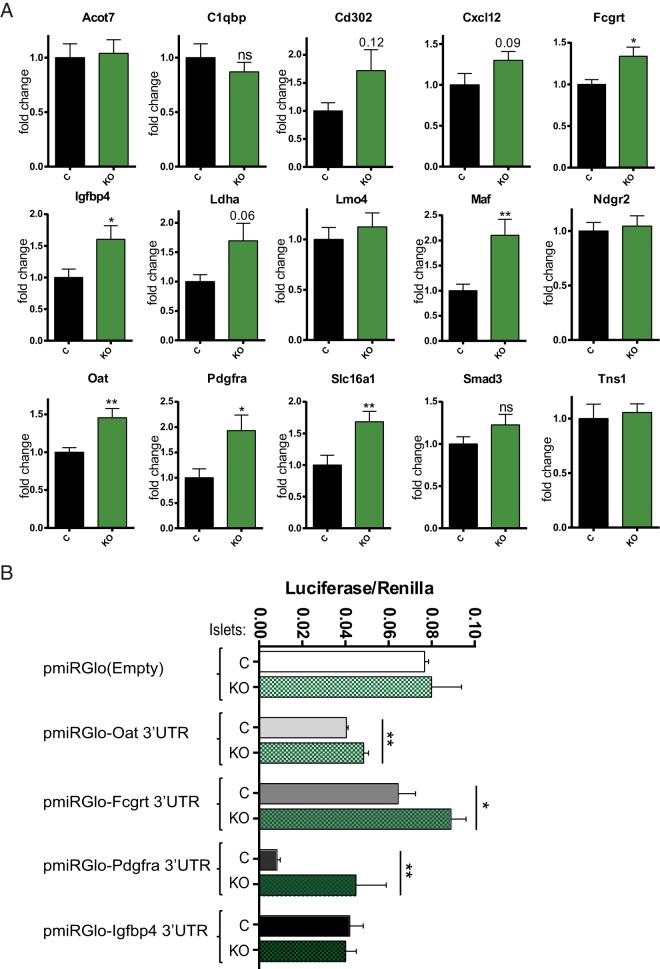
Disruption of miRNA function results in up-regulation of disallowed genes. A, βDicer-null (KO, 7 to 8 weeks old) or control Dicer^flox/flox^ (C) mice were injected with 2 mg of tamoxifen for 5 consecutive days. Two weeks after the last injection, total RNA was extracted from isolated islets and RT-qPCR used to evaluate the expression of the indicated genes (n = 9–13 mice/genotype). B, Sequences coding *Oat*, *Fcgrt*, *Pdgfra*, and *Igfbp4* 3′-UTRs were cloned downstream of the firefly luciferase open reading frame in the plasmid pmirGLO, as indicated. The constructs were transfected into islets extracted 2 weeks after tamoxifen injection of βDicer-null (KO) or control Dicer^flox/flox^ (C) mice, as described under *Materials and Methods*. Data correspond to 2 technical replicates in islets from 3 to 5 different mice/genotype. *, *P* < .05; **, *P* < .01, Student *t* test.

We next used RT-qPCR to measure the expression of putative miRNA target genes in islets from mice isolated 2 weeks after tamoxifen treatment. We reasoned that any changes in gene expression at this time point could be ascribed to a loss of cell autonomous actions of DICER in the β-cell, rather than to indirect effects of hyperglycemia or differences in the cellular composition of islets, in contrast to what may be observed at later stages. We selected 14 disallowed genes for analysis based on their identification in 2 independent studies (*C1qbp*, *Cd302*, *Cxcl12*, *Igfbp4*, *Ldha*, *Lmo4*, *Maf*, *Oat*, *Pdgfra*, *Slc16a1*, and *Smad3*) (6, 7) and/or other criteria including up-regulation in type 2 diabetes (*Acot7*, *Ldha*, and *Pdgfra*) (5). We also explored the regulation of disallowed genes up-regulated upon disruption of AMP-activated protein kinase (AMPK) activity (40) which also included several of the genes listed above (*Acot7*, *Cxcl12*, *Igfbp4*, *Ldha*, *Lmo4*, *Oat*, *Pdgfra*, *Slc16a1*, and *Smad3*) plus *Fcgrt*, *Ndgr2*, and *Tns1*, genes only identified as disallowed by 1 of the 2 studies mentioned above. AMPK-null mice present an alternative model of impaired insulin secretion and loss of β-cell identity, whose islets display defective insulin secretion and up-regulation of disallowed genes (40). Of the 14 disallowed genes studied, 6 (*Fcgrt*, *Igfbp4*, *Maf*, *Oat*, *Pdgfra*, and *Slc16a1*) were significantly up-regulated in βDicer-null islets (Figure 5A).

### Direct action of miRNAs on disallowed genes

S*lc16a1* and *Maf* have previously been shown (31, 41) to be direct miRNA targets in vitro. To determine whether the 4 other candidates may also serve as direct targets for miRNAs, we generated fusion constructs between the respective 3′-UTRs and firefly luciferase (Figure 5B). These constructs were then transfected into islets from wild-type or βDicer-null mouse, with an increase in firefly luciferase activity in the latter, normalized to that of *Renilla* luciferase (independently expressed from the same vector), taken as evidence for miRNA-mediated silencing. 3′-UTRs derived from the *Fcgrt*, *Oat*, and *Pdgfra*, but not *Igfb4*, mRNAs all showed an enhanced ratio of firefly*/Renilla* activity in βDicer-null islets. As expected, firefly*/Renilla* activity remained unchanged when the empty vector was transfected as a negative control.

**Figure 6.**
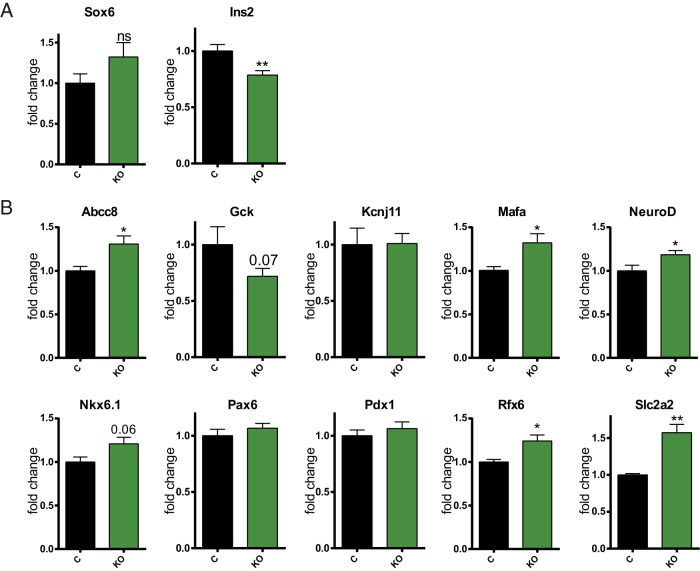
Disruption of miRNA function does not result in impaired expression of β-cell signature genes. A and B, 7- to 8-week-old βDicer null (KO) or control Dicer^flox/flox^ (C) mice were injected with 2 mg of tamoxifen for 5 consecutive days. Two weeks after the last injection, total RNA was extracted from isolated islets and RT-qPCR was used to evaluate the expression of the indicated genes (n = 7–12 mice/genotype). *, *P* < .05; **, *P* < .01, Student *t* test; ns, not significant.

The defects observed in β-cell secretory function could potentially be due to impaired expression of “signature” genes in the β-cell, known to be essential for its differentiation and function (2). Nevertheless, we did not observe a significant reduction in the levels of expression of a number of such genes studied, including the key transcription factors *Pdx1* or *Pax6* as well as others (Figure 6B), in βDicer-null islets. Furthermore, some of these genes were up-regulated upon Dicer depletion, which may suggest that miRNAs also usually restrict their expression. We noted that Melkman-Zehavi et al (16) have previously reported that Dicer depletion causes an increase in the expression of the transcriptional repressor *Sox6*, which in turn resulted in a strong reduction in insulin expression. Contrary to these findings, when *Sox6* was examined 2 weeks after tamoxifen injection, it was not significantly up-regulated in βDicer-null mouse islets (Figure 6A). Therefore, we do not expect missexpression of this transcriptional repressor to contribute to the early impairment in GSIS observed. In line with this finding and at this early stage after Dicer deletion, changes in insulin protein levels were not evident (Figure 2A), and only a small reduction in Ins2 mRNA expression was detected (Figure 6A).

## Discussion

The results provided here support a key role for miRNAs in the maintenance of both the function and survival of mature β-cells and highlight the importance of these species in the suppression, by a direct interaction with the cognate mRNAs, of disallowed genes in these cells.

Pancreatic β-cells are responsible for insulin release and thus are essential for normal blood glucose homeostasis. Although the pathogenic mechanisms underlying type 1 and type 2 diabetes are different, both involve a decrease in β-cell mass and, in the case of type 2 diabetes, a lowered capacity to produce and secrete insulin (42). A loss of the differentiated status (or “identity”) of these cells contributes in many cases to this functional failure (43). Previous studies have shown that when Dicer is depleted in endocrine precursors, α- and β-cells still specify appropriately in the pancreas. However, strong defects in islet morphology and hormone expression occur soon after birth, and there is a severe reduction in pancreatic cell mass (34). Furthermore, depletion of Dicer in all pancreatic cells early during development using PdxCre-mediated recombination leads to gross defects in all pancreatic lineages, with a particularly dramatic reduction in the number of insulin-producing β-cells (23). Nevertheless, β-cell–specific depletion of Dicer during embryonic development only affected islet cell morphology and survival during adult life (24, 44).

Our study is the first to show that survival of β-cells is compromised if miRNA function is specifically depleted in adult mice. Melkman-Zehavi et al (16) have previously generated a model for miRNA depletion in the adult β-cell, by crossing a Dicer1 conditional allele-harboring mouse with RIP-CreER transgenic mice and inducing tamoxifen-dependent recombination in 1- to 5-month-old animals. Contrary to our findings, these authors did not report significant changes in β-cell mass or apoptosis. The apparent discrepancy between the latter result and the findings of the present work may reflect the timing at which the respective studies were performed (3 weeks vs 4 weeks after tamoxifen injection) and the extent of Dicer depletion: we observed a >70% reduction in Dicer mRNA in the whole islets, which, given their cellular composition (2), indicates that the deletion of Dicer occurred in most β-cells. In addition, our experiments were consistently performed in mice aged 7 to 8 weeks vs the broad range of ages (1–5 months) used in the study of Melkman-Zehavi et al (16). Importantly, our data also demonstrate that apoptosis is a likely contributor to the observed β-cell loss. Previous studies in adult mice failed to show apoptosis as a mechanism contributing to β-cell mass reduction, probably because of the technical challenge of detecting a highly dynamic event in which clearance of apoptotic cells might be extremely rapid (24, 25).

However, and in agreement with our results, the Dicer-null mice of Melkman-Zehavi et al (16) presented with hyperglycemia and glucose intolerance as early as 2 weeks after tamoxifen injection. The authors therefore concluded that diabetes developed as a consequence of the strong decrease in pancreatic insulin content that they clearly observed by 3 weeks after tamoxifen treatment. Here, we detected a clear decrease in β-cell insulin content 4 weeks after the injections, but no change in insulin content 2 weeks after tamoxifen administration. Nevertheless, we found that the capacity of βDicer-null islets to secrete insulin in response to high glucose was notably impaired at this stage. These data indicate that an miRNA deficiency affects β-cell secretory function before any decrease in insulin content or β-cell mass occurs.

Kalis et al (24) have previously shown that early (embryonic day 9.5) Dicer deletion in the β-cell results in reduced islet insulin secretion under both low and high glucose once the mice are older than 8 weeks (24). Nevertheless, when normalized for insulin content, strongly reduced in Dicer-null islets, secretion remained unchanged in low glucose and, contrary to our results, even increased in response to high glucose. This apparent discrepancy with our data might have 2 main causes. First, we studied β-cell functionality before changes in β-cell mass or insulin content were apparent. A decrease in β-cell mass could activate compensatory mechanisms that might temporarily lead to enhanced insulin secretion from the remaining β-cells (which, importantly, could be enriched in those that did not undergo *Cre*-mediated recombination). Although the authors failed to detect changes in apoptosis, probably due to technical limitations, they observed a substantial reduction in β-cell mass. Second, we performed Dicer deletion in adult β-cells (7–8 weeks old) vs their depletion during embryonic development. Importantly, miRNA composition dynamically changes during development and maturation (45, 46), such that the precise moment at which Dicer depletion takes place may greatly affect the functional outcome.

### Which specific miRNAs are involved in the effects of Dicer deletion?

To our knowledge, this is the first study to clearly show alterations in GSIS upon general depletion of miRNA species and to demonstrate that this precedes apparent changes in islet insulin content or β-cell mass. A role in β-cell secretory function has already been shown for a number of specific miRNAs. For example, miR-375 affects insulin exocytosis by repressing myotrophin (47), whereas miR-7a modulates GSIS by controlling genes involved in insulin granule fusion (19). Other miRNAs that affect insulin exocytosis include miR-124a, miR-96, and miR-9 (18) (48). On the other hand, miRNAs such as miR-21, miR-34a, or miR-146 modulate apoptosis (21, 22). Overall, insulin secretion may thus be fine-tuned accordingly to the levels of different miRNAs that regulate cell function and mass (49). Which of these or other miRNAs are responsible for the defects in β-cell function and survival observed in our model of Dicer depletion remains to be explored.

Loss of β-cell identity is characterized by increased expression of normally repressed or disallowed genes, such as hexokinase (*HKI-III*) or *Ldha*. Several mechanisms might contribute to β-cell–specific disallowance. So far, only 2 disallowed genes have been determined as direct miRNA targets in vitro. Thus, miR-29 isoforms target *Slc16a1* in islets and therefore might prevent undesired pyruvate-stimulated insulin release (31). Likewise, *Maf* (cMAF), an enhancer of glucagon expression in α-cells (50, 51), is targeted by miR-200c/182/125b in the β-cell line MIN6, potentially restricting β- to α-cell dedifferentiation (41).

Nevertheless, the impact on disallowed gene expression has not previously been assessed in any of the Dicer-null models generated. Using this approach, we confirm first that the miRNA-mediated regulation of both *Slc16a1* and *Maf* occurs in vivo. In addition, we show that the expression of 4 additional disallowed genes (*Pdgfra*, *Oat*, *Fcgrt*, and *Igfbp4*) is silenced by miRNA. Kanji et al (34) have also reported that loss of DICER in NGN3^+^ precursors leads to the up-regulation of a subset of neuronal genes and the loss in binding of the neuronal transcriptional repressor RE-1–silencing transcription factor (REST) to cognate sites. Although a similar (ie, transcription factor-dependent) mechanism may be involved here, *Maf* and *Slc16a1* mRNAs had already been proved to be direct miRNA targets in β-cells in vitro (31, 41). Importantly, we demonstrate direct actions of miRNAs for 3 of the 4 other disallowed genes, *Fcgrt*, *Pgfra*, and *Oat*. In contrast, *Igfbp4* 3′-UTR did not mediate miRNA-dependent repression of luciferase activity, indicating that *Igfbp4* up-regulation most probably occurs indirectly upon miRNA depletion. Nevertheless, we note that miRNAs can also repress their target mRNAs by binding to coding regions (52) or by repressing mRNA translation without apparent changes in mRNA levels.

The up-regulation of 6 disallowed genes correlates with loss of the secretory capacity of the islets, but precedes changes in β-cell mass or insulin content. At this stage, we did not observe a decrease in β-cell signature gene expression, such as *Pdx1* or *Slc2a2*, that could contribute to alter cell function. Indeed, the expression of some of these genes was actually slightly but significantly up-regulated. This is not totally surprising, because miRNAs are capable of simultaneously regulating multiple targets, which can direct or indirectly affect several cellular processes. Further investigation would be required to assess whether these effects occur direct or indirectly as a consequence of miRNA action in our model. Nevertheless, several of these mRNAs, including *Abcc8*, *Mafa*, or *NeuroD*, have already been shown to be subject to direct miRNA-mediated repression under diverse conditions (14, 53, 54).

### What are the roles in β-cell function of specific disallowed genes targeted by miRNAs?

The importance of the silencing of a few of the disallowed genes, such as *Ldha* or *Slc16a1* (MCT-1), has already been demonstrated: their overexpression leads to aberrant fuel signaling (5, 11, 55). On the other hand, *Maf* (also known as v-*Maf* or c-*Maf*) activates glucagon expression in α-cells (57). Klein et al (41) found that *Maf* is repressed by miR-200c, miR-125b, and miR-182. Importantly, *Maf*, while disallowed in β-cells, is highly expressed in α-cells. Conversely, miRNA-200c, niRNA-125b, and miRNA-182 are strongly up-regulated in β-cells, but are poorly expressed in α-cells, presumably allowing the production of cMAF and therefore specifically reinforcing the expression of glucagon (41). Whether loss of Dicer therefore leads to a switch from β- to α-cell fate in the present model is unclear, although it should be emphasized that we did not observe a major change in the number of α-cells per islet.

*Pdgfra* has recently been revealed as a key regulator of age-dependent β-cell proliferation in both human and mouse islets. *Pdgfra* silencing in adult β-cells contributes to restrict proliferation, although the mechanisms involved in its repression remain poorly understood. Although *Pdgfra* has previously been shown to be repressed by miRNAs in other cell types and in cancer-derived cell lines (58, 59), this is, to our knowledge, the first report to demonstrate that miRNAs directly target this mRNA in β-cells. In this context, a means of expanding insulin-secreting β-cells would be highly beneficial in the treatment of diabetes. At present, however, approaches to this have proven challenging and limited (60). Identifying those miRNAs that silence *Pdgfra* in β-cells could therefore contribute to the development of treatments aimed to improve β-cell survival.

*Oat* is an ornithine aminotransferase whose function lies in the glutamate to ornithine conversion pathway. Glutamate has been suggested as an intracellular second messenger that couples glucose metabolism to insulin secretion (61, 62). Thus, *Oat* silencing might prevent the dissipation of this second messenger in β-cells, potentiating secretion. The relevance of *Fcgrt* silencing for β-cell function remains unknown. Neonatal Fc receptor (FcRn) mediates IgG transport across epithelial cells and is involved in antigen presentation (63). We note that although *Fcgrt* is up-regulated in AMPK models and is described as disallowed in the report from Thorrez et al (7), this gene did not appear in our own list (6).

In conclusion, our study further defines the impact of miRNA function in the adult β-cell, showing that miRNA loss first alters β-cell secretory function to eventually result in β-cell death. Most importantly, we have identified 3 disallowed genes that are direct miRNA targets. The identity of the specific miRNAs responsible of the silencing might be worthy of further investigation because manipulation of their expression could contribute to reinforce β-cell identity and function and hence to develop more effective treatments for diabetes mellitus.
